# A computational workflow for proteome-wide analysis of sulfur-containing amino acid distribution in crop proteomes

**DOI:** 10.1016/j.jgeb.2026.100818

**Published:** 2026-09-24

**Authors:** Husam Jamal Omari, Maher Jamal Tadros

**Affiliations:** a Department of Plant Production, Faculty of Agriculture, Jordan University of Science and Technology, Irbid 3030, Jordan; b Department of Natural Resources and Environment and Department of Plant Production, Jordan University of Science and Technology, Irbid 3030, Jordan

**Keywords:** Bioinformatics, Proteome, Amino acids, Methionine, Cysteine, Legumes

## Abstract

Grain legumes are important sources of plant protein for human food and animal feed, but their nutritional quality is often limited by relatively low levels of sulfur-containing amino acids, particularly methionine. Despite their biological and nutritional importance, the distribution of methionine and cysteine across reference proteomes and within protein sequences remains insufficiently characterized. This study developed and applied saa_proteome, a reproducible Python-based framework for proteome-wide, protein-level, and localized analysis of methionine, cysteine, and total sulfur-containing amino acids (S-AA). The framework was applied to the reference proteomes of soybean (*Glycine* max), chickpea (*Cicer arietinum*), white lupin (*Lupinus albus*), and common bean (*Phaseolus vulgaris*). Despite substantial differences in proteome size, methionine ranged only from 2.17% to 2.21%, cysteine from 1.85% to 1.92%, and total S-AA from 4.05% to 4.11%. In contrast, top-ranked proteins displayed pronounced heterogeneity, including cysteine-dominated, methionine-dominated, and comparatively balanced S-AA profiles. Independent rankings using overlapping 100-amino-acid windows identified largely distinct protein sets, with maximum localized enrichment reaching 31% methionine, 38% cysteine, and 38% total S-AA. Highly methionine-enriched regions occurred in diverse predicted protein classes, including transcriptional regulators, membrane-trafficking proteins, and heavy metal-associated proteins. Top-ten protein sets obtained with and without N-terminal methionine removal showed 90% overlap and a Jaccard similarity of 0.82. These findings demonstrate that S-AA composition is stable across the analyzed reference proteomes but highly heterogeneous among proteins and sequence regions. The framework identifies sequence-encoded candidates, although tissue-specific expression and protein abundance remain necessary for evaluating their biological and nutritional relevance.

## Highlights

• S-AA levels were stable across four legume reference proteomes.
• Protein and regional S-AA composition showed strong heterogeneity.
• Sliding windows ranked localized Met, Cys, and total S-AA enrichment.
• Results were robust to N-terminal methionine preprocessing.

## 1 Introduction

Global demand for plant protein-based products is high and is expected to grow significantly over the next decade.^1^ Grain legumes are widely recognized as key staple sources of protein for both human consumption and animal feeding systems.^2^ According to WHO recommendations,^3^ protein intake of approximately 10–15% of total daily energy is generally sufficient to meet adult requirements. In developed countries, these requirements are largely met through consumption of animal-derived proteins, whereas in many developing regions, plant proteins constitute the primary dietary source. Although legumes and other plant proteins are critical for food security, they generally exhibit an imbalanced essential amino acid profile. In particular, they are typically limited in sulfur-containing amino acids, namely methionine and cysteine.^2^, ^4^, ^5^

Methionine and cysteine perform complementary biological functions in plants and animals. Methionine is involved in protein synthesis, serves as a precursor for several essential metabolites, and contributes to growth and developmental processes.^6^, ^7^ The thiol group of cysteine supports disulfide-bond formation, redox regulation, metal coordination, and the synthesis of glutathione and other sulfur-containing metabolites.^8^, ^9^ From a nutritional perspective, however, methionine has received greater attention because it is frequently limiting in legume proteins. In lactating cows, supplementation with methionine and lysine has been shown to increase milk protein yield and overall milk production.^10^, ^11^, ^12^

To meet the increasing demand for methionine in human food and animal feed, numerous studies have explored transgenic overexpression of heterologous methionine-rich proteins to enhance sulfur amino acid content in legumes. However, the effectiveness of these approaches may be constrained by metabolic sulfur availability, protein-folding capacity, genotype-dependent responses, and regulatory considerations.^7^, ^13^, ^14^ In several cases, increases in total seed methionine have been moderate or genotype-dependent. Consequently, the identification and characterization of native methionine- and cysteine-rich proteins within legume proteomes represent a promising strategy for crop improvement through cisgenic approaches, targeted breeding programs, or transgenic expression systems.^15^, ^16^, ^17^

Proteome-wide average composition alone may not capture localized amino-acid enrichment within individual proteins. Compositionally biased regions can contain concentrated or extended enrichment of particular residue types and may have structural or functional significance.^18^ In the case of cysteine, localized enrichment may contribute to redox regulation and metal coordination, including reactive cysteine sites and cysteine-rich metallothioneins.^8^, ^9^, ^19^ Therefore, combining global composition metrics with localized sequence analysis can provide a more informative basis for identifying sulfur-enriched protein candidates and comparing amino-acid allocation patterns across species.

Although numerous computational frameworks are available for proteomics analysis, contemporary plant-proteomics workflows are predominantly mass-spectrometry based and focus on peptide identification, protein quantification, post-translational modifications, localization, and proteome dynamics.^20^, ^21^, ^22^, ^23^ General-purpose bioinformatics libraries such as Biopython provide sequence manipulation capabilities but are not designed for systematic, proteome-wide residue allocation analysis.^24^, ^25^ Consequently, standardized Python-based frameworks for the integrated analysis of global amino acid composition and localized sequence enrichment across complete reference proteomes remain limited.

To address this need, we developed saa_proteome, a modular Python-based framework for proteome-wide analysis of sulfur-containing amino acids. The workflow integrates standardized sequence processing, whole-protein and proteome-level amino-acid composition analysis, and sliding-window quantification of localized methionine, cysteine, and total S-AA enrichment. In the present study, saa_proteome was applied to four legume reference proteomes to characterize global, protein-level, and localized S-AA distribution patterns, identify sulfur-enriched protein candidates, and facilitate standardized cross-species comparisons.

## 2 Methods

### 2.1 Species selection and proteome data sources

Proteome-wide analyses were conducted for four legume crop species: soybean (*Glycine* max), chickpea (*Cicer arietinum*), white lupin (*Lupinus albus*), and common bean (*Phaseolus vulgaris*). Protein sequences were obtained from publicly available UniProt reference proteomes and downloaded in FASTA format on 25 November 2025 (UniProt Consortium; https://uniprot.org/proteomes). The UniProt proteome identifiers were UP000008827 for *Glycine* max, UP000087171 for *Cicer arietinum*, UP000447434 for *Lupinus albus*, and UP000000226 for *Phaseolus vulgaris*. The proteome identifiers, FASTA filenames, NCBI taxonomy identifiers, and source links are provided in Supplementary Material S1.

Each reference-proteome FASTA file was treated as a complete species-specific sequence dataset. All protein records present in the selected files were passed to the computational workflow. No biological filtering was applied on the basis of transcript abundance, protein expression, tissue specificity, subcellular localization, functional category, or annotation status.

### 2.2 Computational framework and software environment

All analyses were performed using the custom Python package saa_proteome version 0.4.1,^26^ developed for reproducible proteome-wide amino-acid composition analysis. The package and associated analysis notebooks were executed in Python 3.13.15 with NumPy 2.4.2, pandas 3.0.1, Biopython 1.86, and Matplotlib 3.10.8. Within the workflow, protein sequences were parsed from FASTA files using Biopython SeqIO, analytical results were organized as pandas DataFrames, and NumPy and Matplotlib supported numerical operations and visualization, respectively.

Species-specific analyses were conducted using identical parameter settings, and the same computational framework was applied under both N-terminal methionine retention and removal conditions. All calculations were performed using full-precision values, with rounding applied only during preparation of tables, figures, and narrative reporting. An overview of the computational workflow is presented in Fig. 1. Species-specific analysis notebooks for the four proteomes under the N-terminal methionine removal and retention conditions are provided in Supplementary Materials S2a–S5b.

**Fig. 1:**
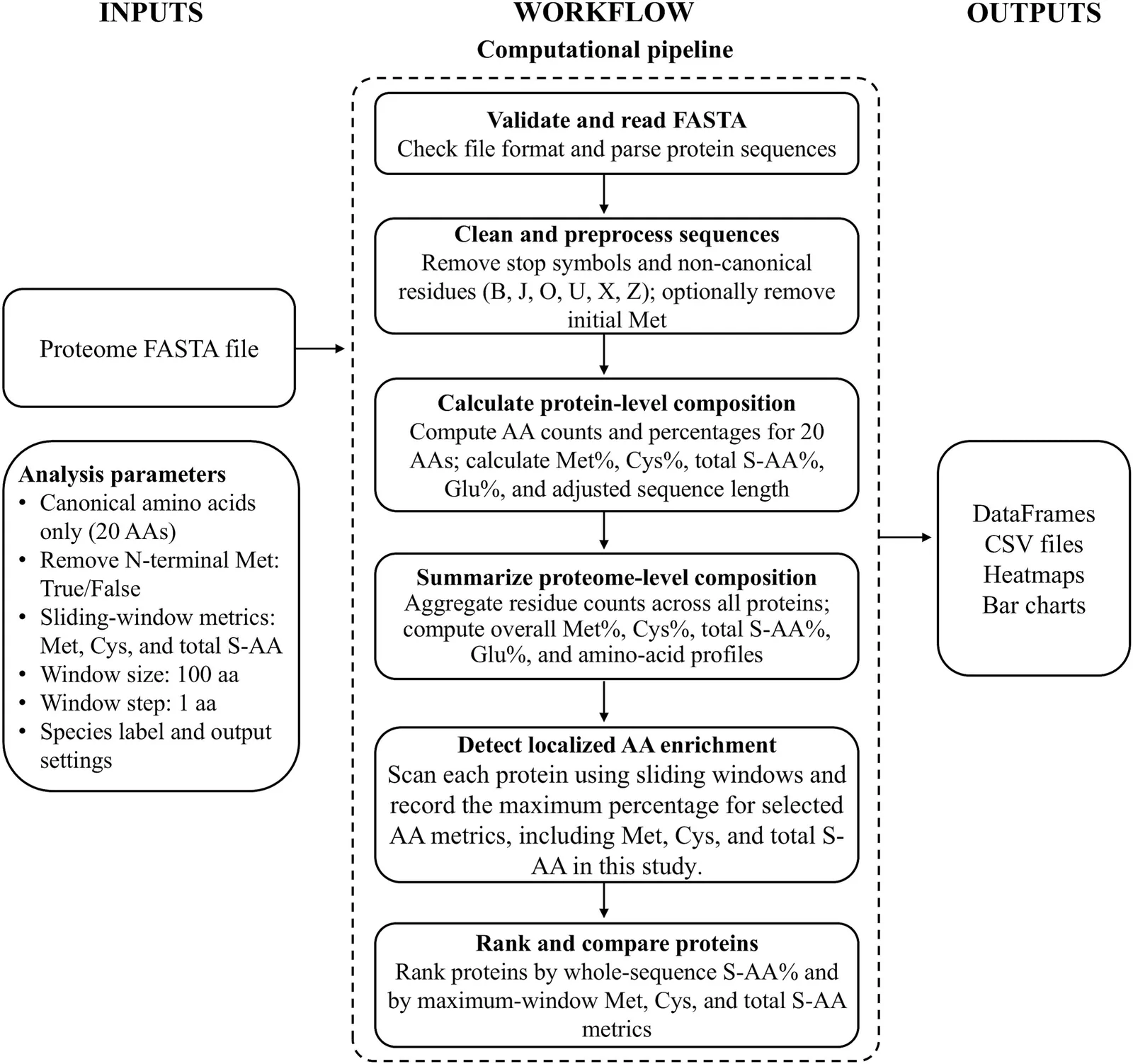
Overview of the computational workflow implemented in the saa_proteome framework.

### 2.3 Sequence validation and preprocessing

Before analysis, each FASTA file was checked for readability, conformity with FASTA structure, and successful sequence retrieval. Protein identifiers were standardized by extracting the UniProt accession from pipe-delimited headers, while the original identifier and full sequence description were retained in the protein-level outputs.

All sequences were normalized by removing surrounding whitespace and converting residues to uppercase. The presence of an N-terminal methionine was evaluated before non-canonical residues were filtered. Under the removal condition (remove_start_m = True), the first residue was removed only when the normalized sequence began with methionine, while under the retained condition, the N-terminus was left unchanged. This order prevented an internal methionine from being misclassified as N-terminal after removal of preceding non-canonical symbols.

Stop symbols (*) and residues outside the 20 canonical amino acids were then removed (canonical_only = True). The excluded non-canonical residue codes were B, J, O, U, X, and Z. Raw, cleaned, and adjusted sequence lengths were recorded for each protein, together with an indicator of whether the N-terminal methionine had been removed. Sequences with an adjusted length of zero were retained for quality-control purposes but excluded from downstream calculations requiring a non-zero denominator.

### 2.4 Protein- and proteome-level amino-acid composition

Amino-acid composition was calculated from the adjusted sequence generated after preprocessing. At the protein level, counts were obtained for each of the 20 canonical amino acids and converted to relative frequencies and percentages using the adjusted sequence length as the denominator. Methionine and cysteine percentages were calculated separately, and total sulfur-containing amino-acid content was defined as their sum. Glutamic acid was quantified using the same procedure and included as a non‑sulfur reference amino acid.

Proteome-level composition was calculated separately for each species by summing residue counts across all eligible processed proteins and dividing each amino-acid total by the total adjusted amino-acid count of the corresponding proteome. Thus, proteome-level percentages were derived from aggregated residue counts rather than from the arithmetic mean of individual protein percentages.

### 2.5 Sliding-window enrichment and protein ranking

The saa_proteome framework supports sliding-window enrichment analysis for individual canonical amino acids and user-defined groups of canonical amino acids, with adjustable window size and step length. In this study, localized enrichment of methionine, cysteine, and total sulfur-containing amino acids was evaluated on the adjusted sequences using overlapping windows of 100 amino acids advanced by one residue at a time. For each protein, the percentage of methionine, cysteine, or their combined total was calculated within every complete window, and the highest observed value was retained as the maximum localized enrichment for that metric.

For adjusted sequences shorter than 100 amino acids, the relevant percentage was calculated across the entire sequence. No averaging or smoothing across windows was applied. Maximum total S-AA enrichment was calculated directly from methionine and cysteine residues occurring within the same window and was not obtained by summing the independently determined maximum methionine and maximum cysteine values.

Proteins were ranked independently using whole-sequence and localized composition metrics. For the whole-sequence comparison, the ten proteins with the highest total S-AA percentage were selected separately for each species under both N-terminal methionine conditions. For localized enrichment, the five proteins with the highest maximum methionine, maximum cysteine, and maximum total S-AA percentages were selected independently for each species. Proteins were sorted in descending order according to the corresponding metric.

### 2.6 Comparative and sensitivity analyses

Outputs from the species-specific analyses were combined into cross-species tables for descriptive comparison of proteome-level composition, protein-level rankings, and localized enrichment metrics. Cross-species descriptive statistics included the arithmetic mean, population standard deviation, range, and coefficient of variation. The population standard deviation was calculated using the number of species as the divisor (ddof = 0), and the coefficient of variation was calculated as the population standard deviation divided by the mean and multiplied by 100. The corresponding proteome-level descriptive comparison workflow is provided in Supplementary Material S9.

Sensitivity to N-terminal methionine removal was evaluated by performing the whole-sequence composition analysis under two preprocessing conditions: retention and removal of the N-terminal methionine when present. Proteome-level methionine, cysteine, total S-AA, and glutamic-acid percentages were compared between the two conditions. The top-ten proteins ranked by whole-sequence total S-AA percentage were also compared within each species to assess changes in protein membership and rank position.

Agreement between the top-ten sets obtained with N-terminal methionine retention and removal was assessed using the number and percentage of shared proteins and the Jaccard similarity index. The percentage overlap was calculated as the number of shared proteins divided by ten and multiplied by 100. The Jaccard similarity index was calculated as the number of proteins common to both sets divided by the total number of unique proteins across the two sets. Rank shifts among shared proteins were examined descriptively. The N-terminal methionine-removed condition served as the primary analysis, while the retained condition was used to determine whether the results were sensitive to N-terminal methionine removal.

### 2.7 Functional annotation of top-ranked proteins

Functional information for the top-ranked proteins was retrieved from the UniProt Knowledgebase (UniProtKB; https://uniprot.org/uniprotkb). Recorded annotations included protein identity, functional description, review status, subcellular location, protein family, conserved domains, and relevant sequence features. UniProtKB review status was recorded to distinguish reviewed Swiss-Prot entries from unreviewed TrEMBL entries.

Annotations were accessed on 18 July 2026 and were used to characterize proteins with high whole-sequence S-AA content or localized methionine, cysteine, and total S-AA enrichment. Functional assignments based primarily on sequence similarity, conserved domains, or automated annotation were interpreted cautiously. The functional comparisons were descriptive only; the frequencies of functional categories among the selected proteins were not statistically compared with their frequencies in the complete proteome.

### 2.8 Code and data availability

The saa_proteome package version 0.4.1, source code, documentation, analysis notebooks, and proteome FASTA files used in this study are available in the project repository: https://github.com/Hussam-Omari/saa-proteome. The package is distributed under the MIT License.

The repository also contains the supplementary workflow description, species-specific notebooks for both N-terminal methionine conditions, cross-species comparison files, the N-terminal methionine sensitivity-analysis notebook and output tables, and the output data dictionary.

## 3 Results and discussion

Proteome-wide analysis of sulfur-containing amino acids was performed using the saa_proteome framework across four legume species: soybean (*Glycine* max), chickpea (*Cicer arietinum*), white lupin (*Lupinus albus*), and common bean (*Phaseolus vulgaris*). The results are presented at three analytical levels: proteome-level composition, protein-level amino-acid distribution, and localized amino-acid enrichment within protein sequences. The primary analyses were conducted after conditional removal of the N-terminal methionine during sequence preprocessing.

Proteome-level summary statistics are presented in Table 1, including the total number of proteins, total adjusted amino-acid count, and the percentages of methionine, cysteine, and total sulfur-containing amino acids (S-AA), and glutamic acid as a non‑sulfur reference. The total number of proteins ranged from 24,828 in *C. arietinum* to 55,852 in *G.* *max*. A similar pattern was observed for the total adjusted amino-acid count, which was highest in *G.* *max*, followed by *L. albus*, *P. vulgaris*, and *C. arietinum*.

**Table 1:** Proteome-level summary statistics for the four legume species.

Species	Total number of proteins	Total number of amino acids	Methionine (%)	Cysteine (%)	Total S-AA (%)	Glutamic acid (%)
*G.max*	55,852	21,638,070	2.19	1.92	4.11	6.33
*C. arietinum*	24,828	10,837,683	2.20	1.88	4.08	6.39
*L. albus*	37,884	14,006,835	2.21	1.85	4.05	6.30
*P. vulgaris*	28,220	11,553,063	2.17	1.92	4.09	6.44

Values correspond to the primary analysis in which the N-terminal methionine was removed when present as the first residue of the cleaned protein sequence.

Despite differences in proteome size, amino-acid proportions varied within narrow ranges across the four species. Methionine ranged from 2.17% to 2.21%, cysteine from 1.85% to 1.92%, total S-AA from 4.05% to 4.11%, and glutamic acid from 6.30% to 6.44%. These results showed that broad proteome-level compositional stability was not restricted to sulfur-containing amino acids. Total S-AA exhibited the lowest coefficient of variation, at 0.49%, followed by methionine at 0.64%, glutamic acid at 0.85%, and cysteine at 1.64% (Table 2). Thus, total S-AA and methionine showed lower relative variability than the non‑sulfur reference amino acid, whereas cysteine showed the greatest relative variation among the four analyzed proteomes. Nevertheless, all coefficients of variation were low, indicating low relative variability in amino-acid composition among the four analyzed reference proteomes.

**Table 2:** Descriptive comparison of proteome-level amino-acid percentages across the four legume reference proteomes.

Amino-acid metric	Mean (%)	SD	Range (%)	CV (%)
Methionine	2.19	0.014	2.17–2.21	0.64
Cysteine	1.89	0.031	1.85–1.92	1.64
Total S-AA	4.08	0.020	4.05–4.11	0.49
Glutamic acid	6.36	0.054	6.30–6.44	0.85

S-AA = sulfur-containing amino acids; SD: population standard deviation (ddof = 0); CV = coefficient of variation, calculated as 100 × SD/mean. Values were calculated from proteome-level outputs generated under the primary analysis setting, with N-terminal methionine removal enabled.

The narrow variability in methionine and total S-AA proportions may be consistent with a combination of general proteome-level compositional constraints and sulfur-specific metabolic regulation. General constraints associated with amino-acid biosynthetic cost and proteome economy may influence amino-acid composition across species.^27^, ^28^ In plants, sulfate uptake and assimilation require metabolically demanding activation and reduction steps, while regulatory mechanisms coordinate sulfur acquisition and allocation to maintain essential cellular functions.^29^, ^30^ However, the similarly low variability observed for glutamic acid suggests that the stability of sulfur-containing amino acids cannot be attributed exclusively to sulfur-assimilation pathways. Broader evolutionary and physicochemical constraints on protein composition may also contribute to the observed cross-species similarity.^27^, ^31^

These findings describe the sequence-encoded amino-acid composition of the four reference proteomes rather than realized amino-acid allocation within particular tissues, developmental stages, or environmental conditions. Because proteins were not weighted by their abundance or tissue-specific expression, the reported percentages do not represent the abundance-weighted contribution of individual proteins to an expressed tissue proteome. Consequently, the observed stability reflects encoded proteomic potential and does not represent quantitative sulfur-amino-acid accumulation in seeds or other plant organs.

The amino-acid composition of the ten proteins with the highest total S-AA percentage was examined under two conditions: retention and removal of the N-terminal methionine (Fig. 2). Under the removed condition, total S-AA content ranged from 21.30% to 27.27% in *G. max*, 17.57% to 23.08% in *C. arietinum*, 22.37% to 37.14% in *L. albus*, and 20.18% to 25.93% in *P. vulgaris*, indicating marked heterogeneity both among and within species. The highest value occurred in *L. albus* protein A0A6A4PC06, which contained 17.14% methionine and 20.00% cysteine, yielding a comparatively balanced total S-AA content. The elevated S-AA percentages reflected distinct compositional patterns, as cysteine and methionine did not contribute uniformly to total S-AA content. Among the 40 proteins analyzed under the removed condition, cysteine exceeded methionine in 35, methionine exceeded cysteine in five, and none contained equal proportions of the two amino acids.

**Fig. 2:**
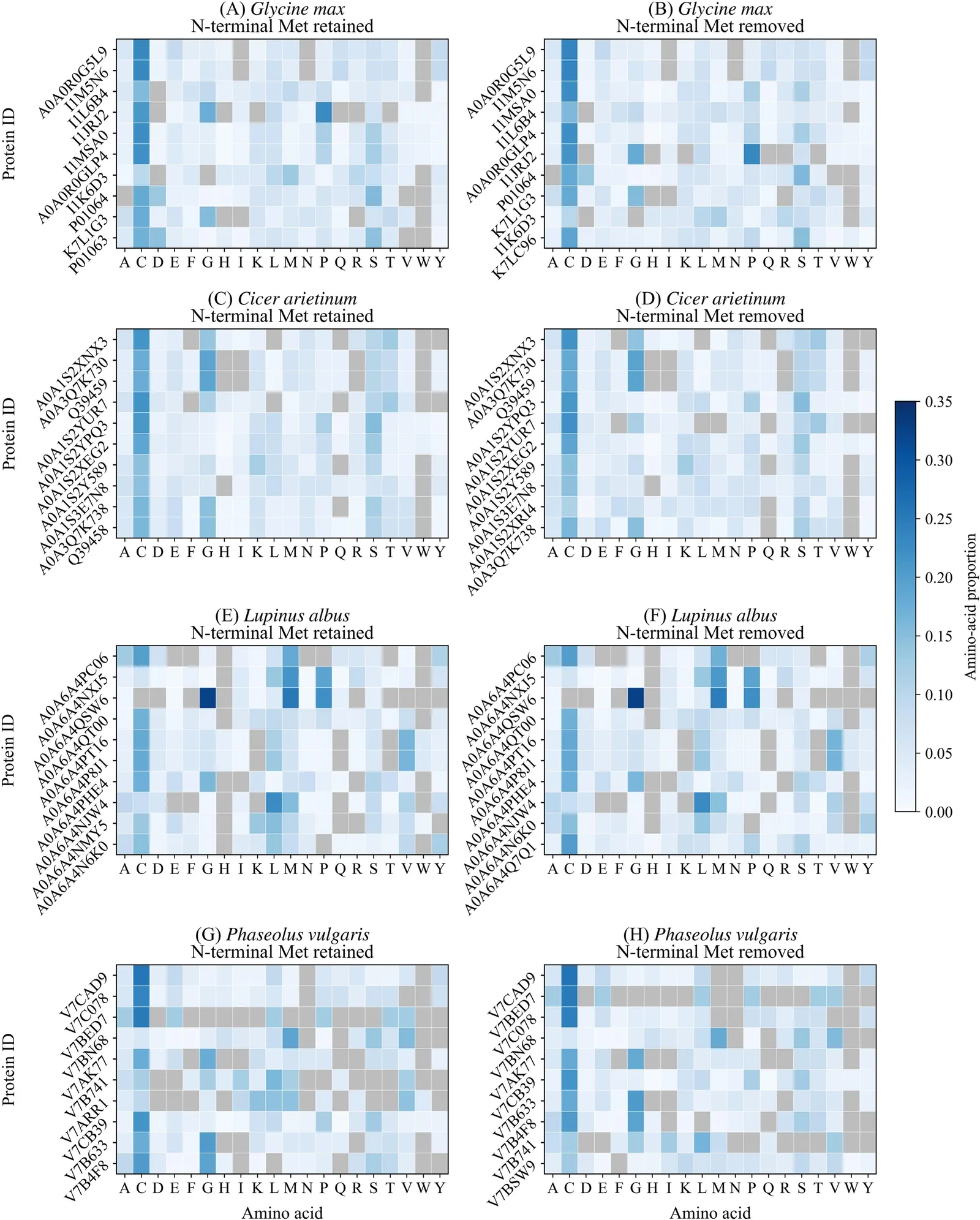
Amino-acid composition heatmaps of the ten proteins with the highest whole-sequence sulfur-containing amino-acid content in each of four legume species under N-terminal methionine retention and removal conditions. Protein sets were selected separately for each preprocessing condition. Gray cells indicate absence of the corresponding amino acid in the sequence.

All ten *C. arietinum* proteins were cysteine-dominated, and nine of the ten *G. max* proteins showed the same pattern. By contrast, *L. albus* displayed the broadest range of compositional patterns, including comparatively balanced, cysteine-dominated, and methionine-dominated proteins. Among the methionine-dominated proteins, A0A6A4QSW6 was especially distinctive, containing 24.60% methionine and no cysteine. *P. vulgaris* likewise included strongly cysteine-dominated proteins, such as V7CAD9, and methionine-dominated proteins, such as V7BN68. Thus, ranking by total S-AA identifies multiple compositional classes, and the principal compositional distinction lies in how total S-AA is partitioned between methionine and cysteine. Because the proteins were selected directly by total S-AA percentage, enrichment in at least one sulfur-containing amino acid is expected from the ranking criterion itself.

UniProtKB-based functional annotation associated these compositional patterns with several distinct protein classes (Supplementary Table S6). The *C. arietinum* set, for example, was dominated by metallothionein-like proteins. Plant metallothioneins are short, cysteine-rich proteins whose thiol groups coordinate metal ions and contribute to metal homeostasis, detoxification, and oxidative-stress responses.^19^ Structural analysis of *C. arietinum* metallothionein-2 further showed that its 14 cysteine residues form metal–thiolate clusters capable of coordinating Zn^2+^ or Cd^2+^, illustrating how the high cysteine content supports metal coordination in this protein family.^32^ Metallothionein-like proteins also occurred among the *G. max*, *L. albus*, and *P. vulgaris* candidates, indicating that this functional class repeatedly contributes to the upper tail of S-AA composition across the four proteomes.

Other cysteine-rich candidates included CYSTM-domain and cysteine-rich transmembrane proteins in *G. max* and *P. vulgaris*. CYSTM proteins are small membrane-associated proteins with conserved cysteine-rich regions implicated in environmental-stress responses and pathogen resistance.^33^, ^34^, ^35^ Their high cysteine content is therefore consistent with their domain architecture, although the physiological roles of individual legume entries should be interpreted cautiously, as several are currently unreviewed or are supported mainly by homology-based annotation.

G-protein γ-domain-containing proteins also recurred in *G. max*, *C. arietinum*, and *P. vulgaris*, with a related protein entering the removed-methionine set in *L. albus*. Plant-specific type-C Gγ subunits can possess extended cysteine-rich C-terminal regions associated with membrane anchoring and signaling, providing a plausible structural basis for their enrichment.^36^, ^37^ Additional species-specific classes included Bowman–Birk protease inhibitors in *G. max* and late-nodulin or incompletely characterized membrane proteins in *L. albus*. These patterns indicate that high S-AA composition can arise in proteins involved in metal binding, membrane association, signaling, protease inhibition, and nodulation-related processes. However, they do not constitute formal functional enrichment, since the selected proteins were not compared with proteome-wide category frequencies.

Methionine-rich proteins were fewer and more heterogeneous. In *L. albus*, A0A6A4QSW6 contained 24.60% methionine, whereas A0A6A4NXJ5 contained 20.80% but remained functionally uncharacterized. In *P. vulgaris*, V7BN68 contained 18.18% methionine and was annotated as an uncharacterized predicted membrane protein. These examples show that compositional enrichment alone does not establish function. Compositionally biased regions may reflect repetitive structure, intrinsic disorder, or specialized interaction properties, but such interpretations require domain-level, structural, and experimental validation.^18^ Particular caution is also required for short fragments, as their calculated percentages can be disproportionately influenced by sequence length.

Removal of the N-terminal methionine caused only limited changes in overall composition and top-ten membership. For each species, nine of the ten proteins were shared between the retained and removed conditions, corresponding to 90% overlap and a Jaccard similarity of 0.82 (Supplementary Table S7). Rank shifts were generally small and were more pronounced in shorter proteins, where removal of one residue represents a larger proportion of the adjusted sequence. Nevertheless, the dominant cysteine-rich, methionine-rich, and mixed profiles remained similar across the paired panels. This sensitivity analysis therefore supports the robustness of the broader interpretation while showing that exact rankings near the top-ten cutoff can depend on N-terminal preprocessing. *In vivo* N-terminal methionine excision depends on the adjacent residue and cellular context; therefore, the two conditions should be viewed as alternative preprocessing assumptions and not as direct representations of the mature state of every protein.^38^

Overall, Fig. 2 demonstrates that the proteins with the highest total S-AA percentages comprise distinct sequence-encoded compositional and functional classes. However, these rankings do not reflect protein abundance in seeds or other tissues. Because plant proteomes vary across organs, developmental stages, environmental conditions, protein turnover, and post-translational processing, reference-proteome composition captures encoded potential, whereas realized S-AA allocation depends on protein abundance and biological context.^22^, ^23^ Tissue-specific transcriptomic or quantitative proteomic data would therefore be required to determine which candidates materially contribute to S-AA accumulation in nutritionally relevant organs.

Localized sulfur-containing amino-acid enrichment was evaluated by independently ranking proteins according to the maximum methionine, cysteine, and total S-AA percentages detected within overlapping 100-amino-acid windows (Fig. 3). The three metrics produced largely distinct protein sets, indicating that localized methionine enrichment and cysteine enrichment represent different compositional patterns. Across the three top-five lists, 11 unique proteins were identified in *G. max*, *C. arietinum*, and *L. albus*, whereas 10 were identified in *P. vulgaris*. No protein appeared in all three lists, and most overlap occurred between the cysteine and total S-AA rankings (Supplementary Table S8). This indicates that the highest localized total S-AA values were generally driven by cysteine-rich regions rather than simultaneous enrichment of both sulfur-containing amino acids.

**Fig. 3:**
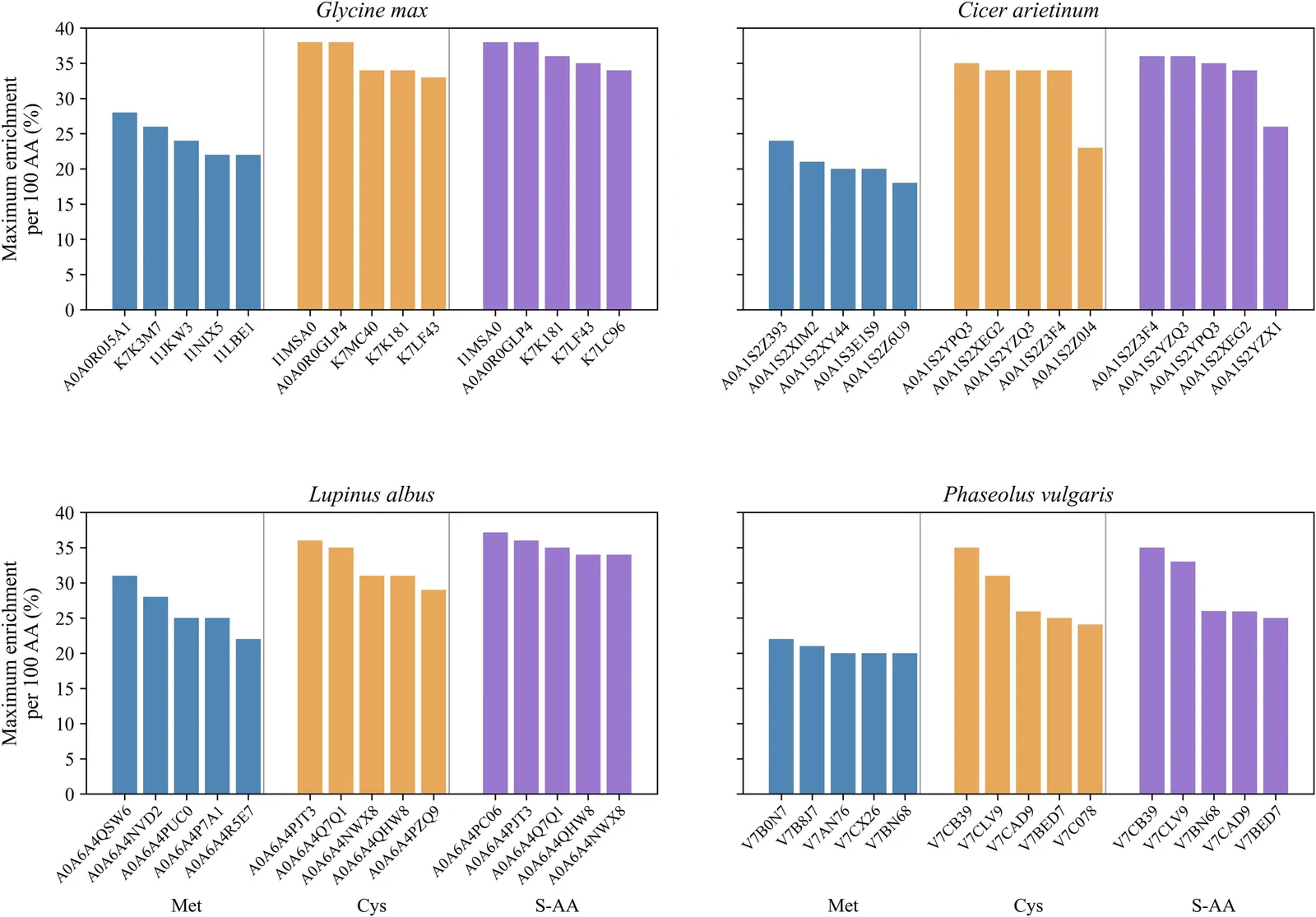
Top five proteins per species ranked independently by maximum proportions of methionine (Met), cysteine (Cys), and total sulfur-containing amino acids (S-AA). Complete 100-amino-acid sliding windows were used for proteins of at least 100 amino acids, whereas the complete adjusted sequence was used for shorter proteins. N-terminal methionine was removed before analysis.

Maximum localized methionine enrichment ranged from 22% to 28% in *G. max*, 18% to 24% in *C. arietinum*, 22% to 31% in *L. albus*, and 20% to 22% in *P. vulgaris*. The highest value occurred in *L. albus* protein A0A6A4QSW6, in which methionine represented 31% of the most enriched window. The complete 126-amino-acid sequence contained 24.60% methionine, indicating that its methionine-rich composition extended across much of the protein rather than being restricted to one narrow segment. Such strong residue-specific enrichment is consistent with compositionally biased or low-complexity sequences, although compositional bias alone is insufficient to establish biological function.^18^ Comparable results were obtained by Stonoha-Arther and Panke-Buisse^4^ using resido, an independently developed R-based package. They reported 25.20% whole-protein methionine and 32% maximum methionine per 100 amino acids for the corresponding *L. albus* collagen-like protein, closely matching the 24.60% and 31% values obtained in this study after N-terminal methionine removal. This close agreement indicates consistency between the two independent analytical implementations.

The other methionine-ranked candidates included predicted FAR1-, bHLH-, UNE10-, and PIF1-like transcriptional regulators, heavy metal-associated isoprenylated plant proteins, and ENTH-domain proteins involved in membrane trafficking (Supplementary Table S8). Several ENTH-domain proteins contained no cysteine but reached maximum methionine values of 21% to 25%, while some transcriptional regulators and heavy metal-associated proteins reached 20% to 28%. Localized methionine enrichment was therefore not confined to storage proteins but occurred across proteins with predicted regulatory, trafficking, and metal-associated functions. Most entries were unreviewed UniProtKB records, however, and their annotations were derived predominantly from sequence similarity, conserved domains, and automated annotation systems. These functional assignments should consequently be interpreted as provisional.^39^, ^40^

Localized cysteine enrichment was generally higher than localized methionine enrichment. The top-five cysteine values ranged from 33% to 38% in *G. max*, 23% to 35% in *C. arietinum*, 29% to 36% in *L.* *albus*, and 24% to 35% in *P. vulgaris*. G-protein γ-domain proteins were the most recurrent category, occupying the first two cysteine ranks in *G. max*, *C. arietinum*, and *L. albus*, and the first rank in *P. vulgaris*. Plant-specific type-C Gγ subunits commonly possess extended cysteine-rich regions associated with membrane localization and heterotrimeric G-protein signaling, providing a plausible structural explanation for their repeated occurrence among the most cysteine-enriched proteins.^36^, ^37^

Alpha/beta-hydrolase-domain proteins formed another recurring cysteine-rich category, particularly in *C. arietinum* and *L. albus* (Supplementary Table S8). Their maximum cysteine percentages reached 29% to 34%, whereas their whole-protein percentages were substantially lower, indicating regional concentration of cysteine. By contrast, the leading G-protein γ proteins were cysteine-rich across larger proportions of their sequences. Thus, similar maximum-window values may reflect either broadly elevated cysteine content or localized enrichment within longer proteins.

The total S-AA ranking largely followed the cysteine ranking. For example, *G. max* proteins I1MSA0 and A0A0R0GLP4 each reached 38% maximum cysteine and total S-AA, while *P. vulgaris* V7CB39 reached 35% for both metrics. In *C. arietinum*, the two highest total S-AA values, both 36%, occurred in alpha/beta-hydrolase proteins. Each protein independently reached maximum cysteine and methionine values of 34% and 5%, respectively. Thus, a high localized total S-AA percentage did not necessarily reflect balanced enrichment of methionine and cysteine. An exception was *L. albus* A0A6A4PC06, which contained 17.14% methionine and 20.00% cysteine and reached 37.14% total S-AA. Similarly, *P. vulgaris* V7BN68 reached 26% total S-AA through a methionine-dominated window containing 20% methionine and 6% cysteine. These examples support retaining separate Met, Cys, and total S-AA rankings because the same aggregate S-AA value can arise from markedly different compositional profiles.

Interpretation of proteins shorter than the 100-amino-acid window requires caution. For these sequences, the complete adjusted sequence was used as the representative window. Accordingly, the 37.14% S-AA value of the 70-amino-acid A0A6A4PC06 and the 25.93% cysteine value of the 54-amino-acid CYSTM protein V7CAD9 represent whole-sequence composition rather than enrichment within a complete 100-amino-acid segment. This limitation is particularly important for the eight-amino-acid fragment V7BED7, for which each residue strongly influences the calculated percentage. Such short sequences should therefore not be interpreted as directly equivalent to proteins evaluated using complete 100-amino-acid windows.

The cysteine-rich *P. vulgaris* proteins V7CAD9 and V7C078 were assigned to the CYSTM family (Supplementary Table S8). CYSTM proteins are small, non-secreted proteins characterized by conserved cysteine-rich transmembrane regions and have been implicated in environmental-stress responses.^34^ Functional studies in *Arabidopsis thaliana* further linked individual CYSTM proteins to salt responses through effects on ion transport and reactive oxygen species homeostasis.^35^ Their identification among the highest localized cysteine candidates is therefore consistent with the established architecture of this protein family, although the functions of the corresponding legume entries remain to be experimentally confirmed.

Overall, Fig. 3 demonstrated that regional sequence composition was substantially more heterogeneous than proteome-wide S-AA composition. Methionine-rich regions occurred across diverse predicted regulatory, trafficking, repetitive, and metal-associated proteins, whereas cysteine enrichment was more consistently associated with G-protein γ proteins, alpha/beta hydrolases, and cysteine-rich membrane proteins. Although these compositional patterns are sequence-encoded, the identified proteins should be regarded as candidates for further validation rather than immediate overexpression targets because their nutritional relevance would also depend on tissue-specific expression, abundance, stability, processing, and sulfur availability.

Plant proteomes vary among organs, developmental stages, environmental conditions, and subcellular compartments.^22^, ^23^ Moreover, sulfur fertilization can alter methionine and cysteine concentrations in soybean seed protein,^5^ while metabolic-engineering studies indicate that methionine-rich protein accumulation may be constrained by sulfur loading, cysteine synthesis, and endoplasmic-reticulum protein-folding capacity.^14^ Therefore, saa_proteome provides a systematic screening framework for localized methionine, cysteine, and total S-AA enrichment, while tissue-specific quantitative and experimental evidence remains necessary for candidate selection.

Alternative approaches address related but distinct aspects of amino-acid distribution. Whole-protein composition analysis measures overall residue abundance but may fail to capture short enriched regions within longer proteins. Sliding-window and compositional-bias methods retain positional information and can identify localized residue-enriched segments.^4^, ^18^ Sliding-window analysis retains positional resolution and identifies localized methionine-, cysteine-, or total S-AA-rich segments, although its results depend on window size, step length, sequence length, and preprocessing. Motif, domain, low-complexity, and structural analyses can assist interpretation, whereas transcriptomic and quantitative proteomic approaches provide an abundance-weighted estimate of realized S-AA allocation in specific tissues or conditions. These methods are therefore complementary: sequence-based analysis identifies encoded compositional potential, while expression-aware approaches identify biologically abundant proteins.

The framework can also identify proteins with low methionine, cysteine, or total S-AA content by reversing the ranking direction. Whole-protein metrics can rank low-S-AA candidates, while sliding-window analysis can identify extended S-AA-depleted regions. This application may support research on specialized dietary protein sources and methionine-restriction models. Interest in such screening is supported by animal studies in which dietary methionine restriction improved ulcerative-colitis-related outcomes and reduced fat deposition through changes in gut microbiota and metabolism, although these effects depended on the restriction level and experimental model.^41^, ^42^ However, sequence composition alone does not establish nutritional suitability, which also depends on protein abundance, digestibility, tissue distribution, processing, and overall amino-acid balance.

## 4 Conclusion

This study presents saa_proteome as a reproducible framework for characterizing sulfur-containing amino-acid distribution across the reference proteomes of four legume species at the proteome, whole-protein, and localized sequence levels. The analysis showed that methionine, cysteine, and total sulfur-containing amino-acid proportions varied within narrow ranges at the reference-proteome level, whereas individual proteins and sequence regions exhibited marked compositional heterogeneity. This contrast shows that proteome-level averages alone can mask biologically and compositionally distinct protein- and region-specific patterns.

Analysis of the top-ranked proteins revealed distinct cysteine-dominated, methionine-dominated, and comparatively balanced compositional profiles, while independent localized rankings identified largely different protein sets for methionine, cysteine, and total S-AA enrichment. Functional annotation further indicated that methionine- and cysteine-rich regions occurred across diverse predicted protein classes rather than a single functional category.

Proteome-level composition patterns and the membership of the top-ten whole-sequence S-AA sets were largely consistent under both N-terminal methionine retention and removal conditions, although exact rankings near the selection threshold changed in some cases, particularly for short proteins. This sensitivity emphasizes the need to report preprocessing assumptions and interpret rankings of short sequences cautiously. However, these results describe sequence-encoded compositional patterns and should not be interpreted as direct estimates of amino-acid accumulation in seeds or other tissues. Accordingly, biological prioritization of sequence-based candidates requires integration with tissue-specific expression or quantitative proteomic evidence and subsequent experimental validation.

Beyond sulfur-containing amino acids, the framework can be adapted to other nutritionally limiting residues, user-defined amino-acid groups, and low-content screening by reversing the ranking direction. It therefore provides a general platform for comparative amino-acid profiling, although biological and nutritional applications require integration with transcriptomic or quantitative proteomic data and subsequent experimental validation.

## Declaration of generative AI and AI-assisted technologies in the manuscript preparation

During the preparation of this work, the authors used ChatGPT (OpenAI) to assist in language refinement and clarity. The authors reviewed and edited the content as needed and take full responsibility for the content of the published article.

## CRediT authorship contribution statement

**Husam Jamal Omari:** Writing – original draft, Visualization, Software, Methodology, Formal analysis, Data curation, Conceptualization. **Maher Jamal Tadros:** Writing – review & editing, Validation, Supervision, Conceptualization.

## Declaration of competing interest

The authors declare that they have no known competing financial interests or personal relationships that could have appeared to influence the work reported in this paper.

## Appendix A Supplementary data

Supplementary material S1 : Workflow description and reference-proteome information.

Supplementary material S2a–S5b : Species-specific analysis notebooks for the four legume proteomes under N-terminal methionine removal and retention conditions.

Supplementary material S6 : Functional annotations of top-ranked whole-sequence S-AA proteins.

Supplementary material S7 : Sensitivity analysis comparing N-terminal methionine retention and removal.

Supplementary material S8 : Cross-species localized methionine, cysteine, and total S-AA ranking and annotation tables.

Supplementary material S9 : Proteome-level descriptive comparison workflow. All analyses were performed using the saa_proteome Python package, version 0.4.1. The source code, documentation, analysis notebooks, and proteome FASTA files are available in the project repository: https://github.com/Hussam-Omari/saa-proteome.
