# Supplementary material for: A computational workflow for proteome-wide analysis of sulfur-containing amino acid distribution in crop proteomes
Source: J Genet Eng Biotechnol. 2026 Sep 24;24(4):100818. doi: 10.1016/j.jgeb.2026.100818 (PMC13634734; doi:10.1016/j.jgeb.2026.100818)
Supplement: Supplementary file 1 [file mmc1.docx]

Supplementary Material S1

A Computational Workflow for Proteome-Wide Analysis of Sulfur-Containing Amino Acid Distribution in Crop Proteomes

This document describes the computational workflow used in this study. Species-specific Jupyter notebooks (Supplementary Materials S2a–S5b) document and reproduce the analyses performed for each crop proteome, while Supplementary Material S9 provides the proteome-level descriptive comparison. The notebooks use the custom Python library saa_proteome, developed for proteome-wide amino-acid composition analysis with particular emphasis on methionine and cysteine.

The workflow begins with validation and parsing of species-specific FASTA files obtained from the UniProt reference proteomes listed in Table S1. Protein sequences are normalized before analysis. When remove_start_m=True, an N-terminal methionine is removed only when it occurs as the first residue of the normalized original sequence; optional canonical-residue filtering is then applied to retain the 20 standard amino acids and exclude noncanonical symbols. N-terminal methionine retention was evaluated as a sensitivity condition because actual N-terminal methionine excision depends on biological and sequence context and cannot be inferred from reference-proteome sequences alone. All preprocessing steps are explicitly parameterized and reproducible.

Protein-level outputs include raw, cleaned, and adjusted sequence lengths; preprocessing flags; methionine, cysteine, and combined sulfur-containing amino-acid (S-AA) percentages; and related composition descriptors. Proteins can be ranked by any numerical metric, and defined subsets, including the proteins with the highest total S-AA percentages, can be selected for downstream analysis.

Localized enrichment is quantified using overlapping sliding windows. For each protein, the workflow calculates the maximum methionine, cysteine, and combined S-AA percentages observed within windows defined by the selected window_size and window_step parameters. In this study, 100-amino-acid windows with a one-residue step were used for the localized-enrichment analysis.

Amino-acid composition matrices quantify the relative frequency, from 0 to 1, of each canonical amino acid within each selected protein. Counts and percentages are also supported. The analytical functions return structured numerical outputs, including pandas DataFrames, summary statistics, ranking tables, and visualization-ready matrices. Graphical representation is performed at the notebook level using standard visualization libraries, allowing adjustment of color scales, resolution, and figure layout.

Intermediate and final analytical tables are retained as pandas DataFrames to facilitate inspection, reuse, and export, while figures are generated and saved separately. Together, the saa_proteome library and the accompanying notebooks provide a transparent and reproducible record of the complete pipeline, from raw proteome sequences to proteome-level summaries, ranked S-AA-rich proteins, localized-enrichment profiles, and associated visual outputs.

**Table S1.** UniProt reference proteomes and corresponding FASTA files used for the comparative analysis of sulfur-containing amino-acid distribution in four legume species.

| **Species** | **Common name** | **UniProt proteome ID** | **NCBI taxonomy ID** | **FASTA filename** |
| --- | --- | --- | --- | --- |
| *Glycine max* | Soybean | UP000008827 | 3847 | [UP000008827_3847.fasta](https://www.uniprot.org/proteomes/UP000008827) |
| *Cicer arietinum* | Chickpea | UP000087171 | 3827 | [UP000087171_3827.fasta](https://www.uniprot.org/proteomes/UP000087171) |
| *Lupinus albus* | White lupin | UP000447434 | 3870 | [UP000447434_3870.fasta](https://www.uniprot.org/proteomes/UP000447434) |
| *Phaseolus vulgaris* | Common bean | UP000000226 | 3885 | [UP000000226_3885.fasta](https://www.uniprot.org/proteomes/UP000000226) |
